# Influence of Transition-Metal Order on the Reaction Mechanism of LNMO Cathode Spinel: An *Operando* X-ray Absorption Spectroscopy Study

**DOI:** 10.1021/acs.chemmater.2c01360

**Published:** 2022-07-06

**Authors:** Marcus Fehse, Naiara Etxebarria, Laida Otaegui, Marta Cabello, Silvia Martín-Fuentes, Maria Angeles Cabañero, Iciar Monterrubio, Christian Fink Elkjær, Oscar Fabelo, Nahom Asres Enkubari, Juan Miguel López del Amo, Montse Casas-Cabanas, Marine Reynaud

**Affiliations:** † Center for Cooperative Research on Alternative Energies (CIC energiGUNE), Basque Research and Technology Alliance (BRTA), Alava Technology Park, Albert Einstein 48, 01510 Vitoria-Gasteiz, Spain; ‡ Inorganic Chemistry Department, Science and Technology Faculty, Basque Country University (UPV/EHU), 48940 Leioa, Bilbao, Spain; § Haldor Topsoe A/S, Haldor Topsøes Allé 1, 2800 Kgs. Lyngby, Denmark; ∥ Institut Laue Langevin, 38042 Cedex Grenoble, France; ⊥ Ikerbasque - Basque Foundation for Science, Maria Diaz de Haro 3, 48013 Bilbao, Spain

## Abstract

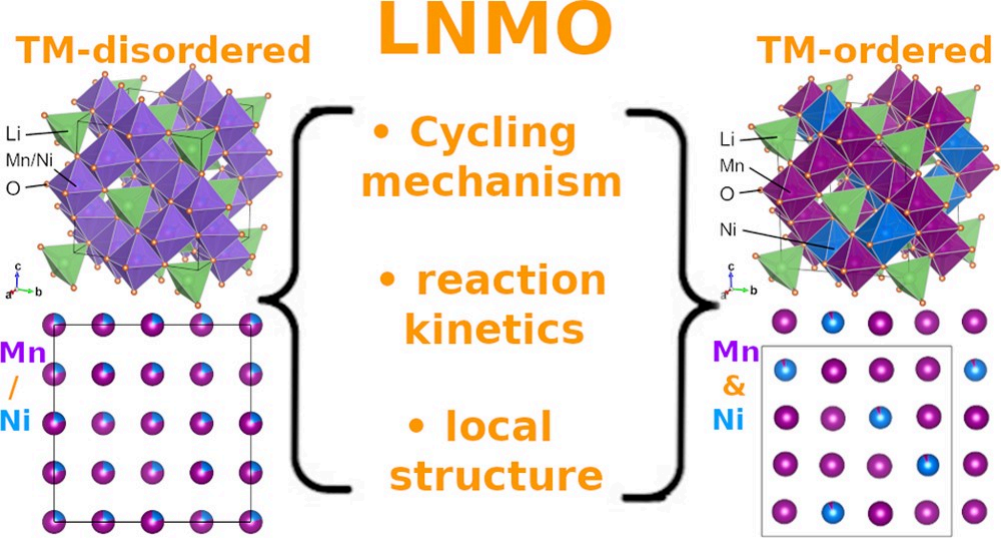

An *operando* dual-edge X-ray absorption spectroscopy on both transition-metal ordered and disordered LiNi_0.5_Mn_1.5_O_4_ during electrochemical delithiation and lithiation was carried out. The large data set was analyzed via a chemometric approach to gain reliable insights into the redox activity and the local structural changes of Ni and Mn throughout the electrochemical charge and discharge reaction. Our findings confirm that redox activity relies predominantly on the Ni^2+/4+^ redox couple involving a transient Ni^3+^ phase. Interestingly, a reversible minority contribution of Mn^3+/4+^ is also evinced in both LNMO materials. While the reaction steps and involved reactants of both ordered and disordered LNMO materials generally coincide, we highlight differences in terms of reaction dynamics as well as in local structural evolution induced by the TM ordering.

## 1 Introduction

**Figure 1:**
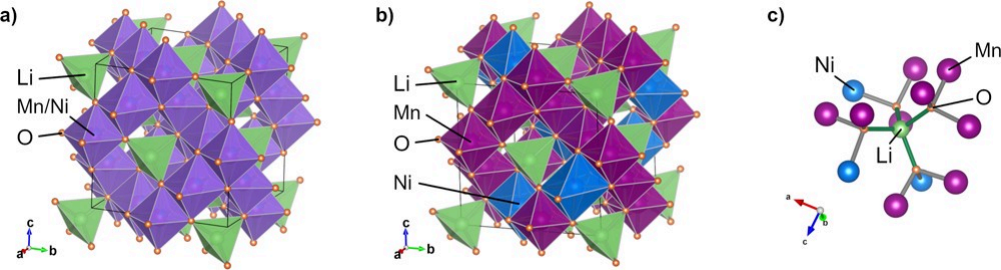
Structural models of the (a) TM-disordered spinel LNMO, described in a *Fd*3̅*m* unit cell, and (b) TM-ordered spinel LNMO, described in a *P*4_3_32 unit cell. Orange balls represent the oxygen atoms; Li are located at the center of the green tetrahedron; in (b), Ni and Mn occupy the center of the blue and purple octahedra, respectively, while in (a) they are randomly distributed in the center of the violet octahedra. (c) Local environment of Li (green ball), surrounded by 4 oxygen atoms (orange balls), 3 Ni (blue balls), and 9 Mn (purple balls) in the perfectly TM ordered spinel.

LiNi_0.5_Mn_1.5_O_4_ (LNMO) is a promising cathode material for next-generation lithium ion batteries, primarily thanks to its favorable structural and compositional properties. Its stable spinel structure and transition-metal stoichiometry allow for fast Li uptake, which enables high rate capability, and elevated operating voltage (≈4.7 V vs Li^+^/Li) yielding to high cell energy (≈ 650 Wh kg^–1^ at the materials level). Moreover, it is intrinsically free of expensive and ethically burdened Co and makes use of a greater share of its structural Li than prevalent NMC, resulting in favorable price competitiveness.^1^ LNMO is formed out of a cubic close-packed array of oxygen atoms, where the transition metals (TMs) occupy half of the octahedral sites formed by the oxygen sublattice and give rise to a stable 3D framework of edge-sharing MO_6_ octahedra, depicted in Figure 1. Li atoms occupy 1/8 of the tetrahedral sites of the structure. The LiO_4_ tetrahedra share their four vertices with MO_6_ octahedra and their four faces with vacant octahedral sites, which enables facile 3D mobility of the Li^+^. The synthesis parameters have been reported to govern the ordering of the TMs.^2,3^ In the disordered LNMO (Figure 1a), the Ni and Mn atoms are randomly distributed on the 16d sites of the *Fd*3̅*m* cubic unit cell, while Li atoms occupy the 8a sites. In the ordered LNMO (Figure 1b), obtained at higher synthesis temperature, Ni and Mn atoms order at 4b sites and 12d sites of the *P*4_3_32 cubic cell, respectively, and Li atoms are located at 8c sites.^4^ Because of distinct synthesis steps, the disordered spinel-based materials are prone to contain a Ni-rich rock-salt phase impurity. This leads to the formation of Mn^3+^ in the *Fd*3̅*m* spinel phase and results in Mn redox activity as well as additional charge carriers.^2^

Great effort has been dedicated to elucidate the effect of different physicochemical properties, such as particle shape and size,^5^ exposed facets,^6^ oxygen,^7^ and TM stoichiometry^2,8,9^ as well as degree of TM ordering,^10,11^ on its electrochemical performance. The latter has particularly been the focus of debate, and estimations regarding its impact on the cycling performance are conjectural. The majority of studies have claimed superiority of the disordered *Fd*3̅*m* phase,^12−15^ but this view has been challenged repeatedly.^7,16−18^

Moreover, the underlying redox mechanism and the role of TM ordering within remain to some parts elusive. It is widely agreed that the main redox activity relies on the Ni^2+/4+^ redox couple, and Mn only sparsely contributes via Mn^3+/4+^.^19^ It has been proposed that in the first part of the charge reaction the TM ordered LNMO undergoes a two-phase transformation, while disordered LNMO a solid solution (single-phase behavior).^4,10^ This difference is also reflected in the electrochemical signature by a slightly elevated insertion/deinsertion potential for the ordered phase as well as a less pronounced step between the two Ni plateaus.^5,20,21^ For the second part of the charge reaction, a mutual biphasic reaction has been reported. Phase evolution has been tracked by *operando* X-ray and neutron diffraction (XRD and NPD) studies, providing indirect insights on the redox mechanism, based on changes of the lattice parameters.^22−25^ Local structural evolution has been monitored with Raman^26−28^ and nuclear magnetic resonance (NMR)^29,30^ spectroscopic studies. Similar to XRD, Raman allows assertion of the TM oxidation state in this case via the TM–O or indirectly via Li–O bond strength.^31^ However, those *in situ* Raman experiments are plagued by attribution ambiguity and interfering electrolyte signals which strongly limit the amount of significant information that can be extracted. X-ray absorption spectroscopy (XAS) is the method of choice for obtaining direct insights into element specific local and electronic structural evolution upon electrochemical cycling.^32^ In soft XAS studies, the redox-active 3d orbitals of TM in LNMO have been probed but, because of experimental constraints, are limited to *ex situ* measurements with few data points and to a surface-confined probing depth.^20,33−35^ Rana et al. have performed bulk sensitive hard XAS under cycling conditions on the partially TM-ordered *P*4_3_32 phase, allowing them to portray quantitative phase evolution as well as to monitor local structural changes. Unfortunately they only acquired a very limited amount of data points during one full electrochemical cycle, leaving an incomplete picture.^36^ Arai and co-workers compared X-ray absorption near-edge structure (XANES) and XRD to investigate the kinetics of phase transitions in disordered LNMO, concluding that XANES reveals a more direct response while XRD is delayed due to time required for domain growth and is hence less suitable to study such reaction dynamics.^23^

Despite all the above-mentioned efforts, none of the up-to-date available studies provide a holistic picture of the TM redox activity and local structural evolution in TM ordered and disordered LNMO under realistic cycling conditions. Here we aim to close this gap by presenting for the first time a dual-edge XAS study under *operando* conditions on both ordered and disordered LNMO spinel phases.

## 2 Experimental Section

### 2.1 Material Characterization

Raman spectra of the material were recorded with a Renishaw spectrometer (Nanonics Multiview 2000) operating with an excitation wavelength of 532 nm. Spectra were acquired with 15 s of exposition time of the laser beam to the sample.

Micrographs were taken on a Thermo Fisher Quanta 200 FEG high-resolution scanning electron microscope (SEM). The working voltages of the Quanta 200FEG 20 kV and Everhart–Thornley detector (ETD) were used for imaging. Particle sizes were evaluated with a MasterSizer 3000 (Malvern Panalytical, Netherlands).

The samples were analyzed with inductively coupled plasma atomic emission spectroscopy (ICP-AES) using a Horiba Ultima 2 (Jobin Yvon, Longjumeau, France) in conjunction with a AS500 autosampler and Activanalyst software (ver. 5.4). The ICP-AES operating conditions were as follows: 1.0 kW of RF power, 13 L min^–1^ of a plasma-gas flow rate, 0.2 L min^–1^ of a sheath-gas flow rate, and 0.25 L min^–1^ of a nebulizer-gas flow rate. Solutions were introduced into the plasma torch by using nebulizer and a cyclonic type of spray chamber at a flow rate of 0.87 mL min^–1^. Calibration solutions were prepared by using a commercial calibration standards of Li, Ni, and Mn (Scharlab, Barcelona, Spain) at concentration of 1000 mg L^–1^. Nitric acid (69%, analytical grade), hydrochloric acid (37%, Ultratrace from Scharlab, Barcelona, Spain), and Ultrapure water from Fischer Scientific (Waltham, MA) were used for dilutions. The most prominent analytical lines of Li 670.784 nm, Ni 216.556 nm, and *M*_n_ 257.610 nm were selected for measurements. Concentrations of these elements were quantified by using the four-point external calibration curve within the concentration range 0.01–100 mg L^–1^.

Solid-state nuclear magnetic resonance and ^7^Li magic angle spinning solid-state nuclear magnetic resonance (MAS NMR) experiments were performed on a Bruker 300WB spectrometer charged to a field of 4.69 T equipped with a standard 1.3 mm MAS probe. Spinning frequencies were set to 50 kHz. A rotor synchronized spin-echo pulse sequence was used with typical 90° and 180° pulses of 1.3 and 2.6 μs, respectively. A recycle delay of 0.5 s was used, and around 1K scans were typically acquired in a ^7^Li NMR experiment. The spectra were referenced to a 1 M solution of LiCl. The spectra were analyzed and deconvoluted by using the Dmfit software package.

Neutron powder diffraction (NPD) of pristine TM disordered (LNMO-D) and TM ordered (LNMO-O) powder was performed by using the D1B diffractometer at the Institute Laue-Langevin (ILL), Grenoble, France, with a wavelength of 1.288 Å.^37^ The sample was mounted on a 6 mm diameter vanadium sample holder and placed on a carousel which allows automatic sample change. The neutron diffraction patterns were collected at room temperature with a high statistic to enhance the data accuracy. Rietveld analysis was performed by using the FullProf suite.^38^ Structural models were constructed by using the VESTA software package there within.

### 2.2 Electrode Formulation and Cell Assembly

For the *operando* experiments LNMO-D and LNMO-O, provided by Haldor Topsoe, were mixed in a NMP-based slurry together with PVdF binder (Kynar HSV900; Arkema, France) and carbon additive (C65; Imerys, Switzerland) in a ratio of 93:3:4 and deposited on single-side carbon-coated Al foil (Armor, France) with active material loading around 10.6 mg cm^–2^. For the laboratory cycling experiment a ratio of 86:8:8 and a loading of 6.1 mg cm^–2^ were used. After cutting, pressing, and drying, electrodes were mounted in CR2032 coin cells for laboratory cycling or in specially designed *in situ* cell for spectroscopic measurements, recently described elsewhere.^39^ The cells were assembled in an argon-filled glovebox (≤0.1 ppm of H_2_O and O_2_) with a LNMO-O or -D positive electrode, a quartz fiber separator (QM-A; Whatman), and a 16 mm diameter lithium disc counter electrode by using LP30 electrolyte. Galvanostatic cycling with potential limitation was performed by using a Bio-Logic ST-150 potentiostat at a C/*n* rate (expressed as 1 mol of Li reacted in *n* hours per mole of LNMO). The electrochemical cycling during *operando* measurements was performed at a C/10 rate within the voltage window of 3.5–4.9 V vs Li^+^/Li; for the second charge the cutoff voltage was raised to 5.0 V vs Li^+^/Li. The laboratory cycling experiments were done at C/20 with a voltage range of 3.5–4.8 V vs Li^+^/Li.

### 2.3 Operando Dual-Edge X-ray Absorption Spectroscopy (XAS)

XAS measurements at the Ni and Mn K-edge were performed in transmission mode at the CLAESS beamline of ALBA synchrotron, Barcelona, Spain. A focusing double-crystal silicon (311) monochromator was used. The *in situ* cell was placed between first and second ionization chamber. The beam size was adjusted to 0.6 × 0.6 mm^2^ (*V* × *H*). XAS spectra were continuously acquired during 1.5 electrochemical cycles alternating every 15 min between the two transmission metal edges (Ni and Mn). For energy calibration TM reference foils placed between the second and third ionization chambers were used. In the extended X-ray absorption fine structure (EXAFS) region data were acquired up to *k* = 16 and 17 Å^–1^ for Ni and Mn, respectively.

### 2.4 Chemometric Data Analysis

The complete *operando* XAS data sets comprising more than 1200 spectra were analyzed by combining principal component analysis (PCA) and multivariate curve resolution-alternating least squares (MCR-ALS) analysis. For more details about the application of these methods please refer to a recent review.^40^ The MCR-ALS analysis for XAS data set was performed with the following constraints: non-negativity of the concentration of the components and closure (sum of the components’ concentrations equal to 100%) as well as a single component at the pristine state. The reconstructed pure spectral components were subsequently fitted in a traditional way, described below.

### 2.5 EXAFS Fitting

The reconstructed pure spectral components were extracted and fitted using the IFEFFIT software package.^41^ The Fourier transform of EXAFS oscillations with different *k* weights was performed in the *k* range from 2.5 Å to 12.0 and 13 Å^–1^ for Mn and Ni, respectively. Fitting was performed in the *R* range from 1.4 to 4.9 Å (not phase corrected) by using *k*^1^, *k*^2^, and *k*^3^ weights. EXAFS amplitudes and phase shifts were calculated by the software package FEFF starting from the lattice parameters of the corresponding LNMO phase ICSD90650 and ICSD70045. Interatomic distances (*R*) and the Debye–Waller factors (σ^2^) were calculated for all paths included in the fits. For the outer shells Ni–O4 and Ni–O5 only single scattering contributions were considered; hence, coordination numbers should be taken with care. To reduce correlation, their sum of coordination numbers was kept fixed and the mutual Debye–Waller factor was imposed.

## 3 Results

### 3.1 Material Characterization

Both types of LNMO, the transition metal ordered (LNMO-O) and transition metal disordered (LNMO-D), were examined regarding their morphological, long-range, and local and microstructural and electrochemical properties. The SEM micrograph presented in Figure S1a shows a comparison of the two LNMO samples revealing that both materials have a similar morphology, composed of very homogeneous secondary spherical particles with an approximate diameter of 6.9(2), 11.2(7), and 18(2) μm for LNMO-D and 6.8(4), 14(3), and 23(5) μm for LNMO-O for Dv10, Dv50, and Dv90, respectively. As can be seen in the micrographs, these secondary spherical particles are composed of smaller polygonal primary particles with an approximate size of 1–2 μm.

Electrochemical cycling of LNMO-D and LNMO-O under laboratory conditions vs Li metal in half-cell coin cells at C/20 reveals salient differences between their electrochemical cycling curves (see Figure S1b). Although in both samples a minority redox contribution around 4.1 V vs Li^+^/Li which is attributed to the Mn^3+/4+^ redox couple can be observed in the charge reaction, it is clearly less pronounced in the LNMO-O. Upon continuation of charge reaction, this is followed by an extended two-step plateau centered at around 4.7 V vs Li^+^/Li for LNMO-D, which is attributed to the Ni^2+/4+^ redox couple. In LNMO-O, this plateau is upshifted by 0.05 V vs Li^+^/Li, and the step height between the two steps is reduced.^5,19^ Despite these differences observed in the electrochemical signature, a similar reversible capacity of 126 mAh g^–1^ is obtained for both samples in the first cycle with a Coulombic efficiency of ≈89%.

**Figure 2:**
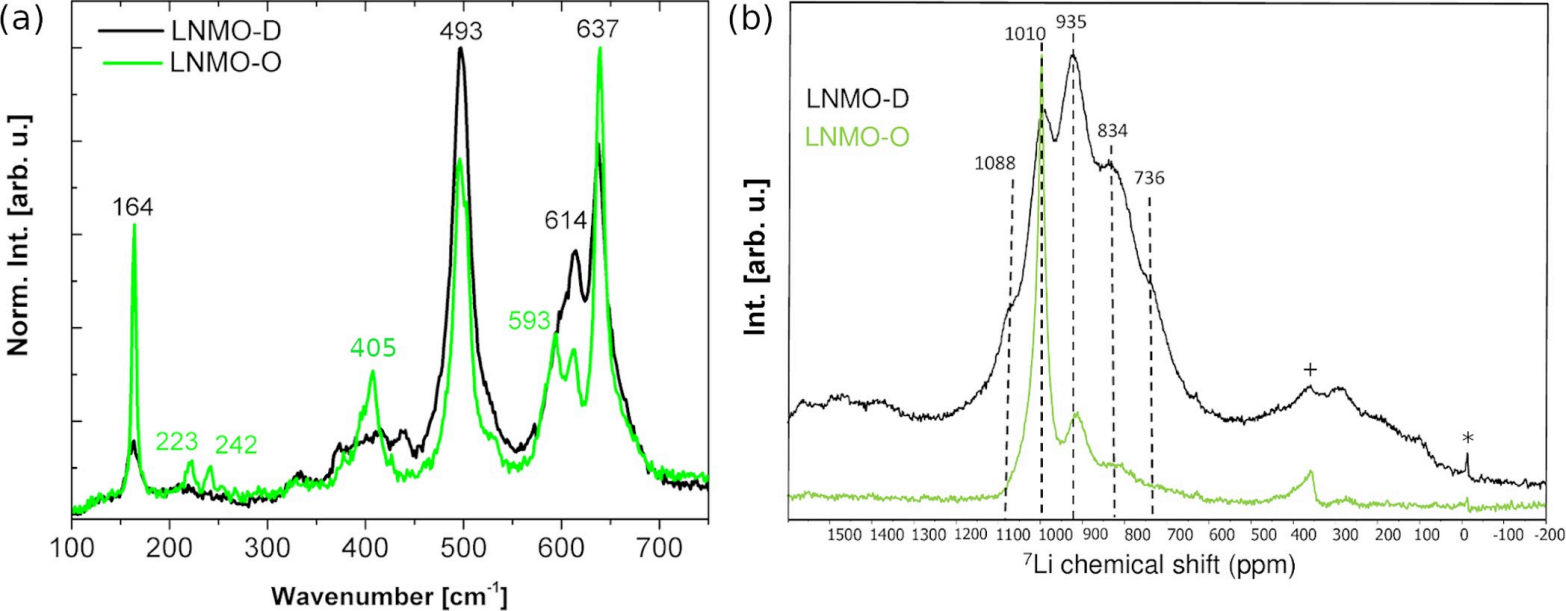
(a) Normalized Raman spectra and (b) ^7^Li MAS NMR spectra of the samples LNMO-O and LNMO-D. NMR shifts are indicated as dashed lines at the approximate center of mass of each resonance. Rotational sidebands appear as echoes of the center lines at a distance in the spectrum of 50 kHz (MAS frequency) and are denoted by (+). Minor signals are observed at 0 ppm (∗) which are assigned to lithium-containing diamagnetic salts.

ICP-AES measurements revealed that both samples have a slightly Ni-deficient stoichiometry with Ni to Li ratio of ≈0.45. Such a deviation has been previously linked to the formation of Mn^3+^, which ensures high electronic conductivity.^5^ Raman spectroscopy allows a facile and reliable discrimination between ordered and disordered LNMO spinel phase thanks to its sensitivity to the local symmetry. The similarities and differences in local structure between the two materials can be appreciated in Raman spectra (see Figure 2a). While the main spectral features of F_2g_ and A_1g_ at approximately 493 and 637 cm^–1^ are mutual, the additional peaks at 223, 242, and 593 cm^–1^ are unique features of the TM ordered phase.^10^ The stronger *E*_g_ peak for the LNMO-O around ≈400 cm^–1^, attributed to Ni^2+^–O stretching mode, further substantiates the attribution of LNMO-D and LNMO-O to the *Fd*3̅*m* and *P*4_3_32 spinel phase, respectively.^4^

The ^7^Li solid-state NMR (ssNMR) spectra of LNMO-O and LNMO-D are presented in Figure 2b. ssNMR can provide detailed insights into the local environments of the nuclei under investigation (Li in this case), even in highly disordered systems. In the case of paramagnetic cathode materials, ssNMR spectra are mainly determined by paramagnetic shifts and broadening, where paramagnetic shifts are the result of the through-bond delocalization of the unpaired electron densities from the metal orbitals into the Li sites.^42^ This additive property results in NMR signals that are strongly displaced from the chemical shift regions normally observed in diamagnetic materials (±10 ppm for ^6/7^Li NMR). Paramagnetic shifts are therefore very useful in the quantitative characterization of metal dispositions around each Li site. In the case of the LNMO spinel structure, shifts depend mainly on the Ni/Mn distribution around lithium as well as on the oxidation state of Mn.^2,43,44^

According to previous studies, the isotropic resonances from the spinel LiNi_0.5_Mn_1.5_O_4_ usually appear between 700 and 1100 ppm for both ordered and disordered materials.^45,46^ In LiNi_0.5_Mn_1.5_O_4_, the first coordination sphere around Li contains 12 transition metal (TM) cations which are connected to Li via Li–O–TM bonds (see Figure 1). In the case of the perfectly ordered spinel, 3Ni^2+^ and 9Mn^4+^ cations are located in the first coordination shell, namely Li(O–Ni^2+^)_3_(O–Mn^4+^)_9_, which is depicted in Figure 1c. This specific coordination is expected to result in a single resonance in the ^6/7^Li NMR spectra, reflecting a unique lithium environment in the crystal. The dominant sharp signal at 1010 ppm in Figure 2b for the LNMO-O reflects the prevalence of a single environment in agreement with previous reports.^29,45^ The presence of additional signals in the LNMO-O spectrum can be explained by small deviations of the Mn:Ni ratios in the first coordination shell of some of the lithium population.^2^ In this regard, the small resonance observed in the LNMO-O spectrum (Figure 2b) at 935 ppm is in agreement with a Mn-rich environment and the broader component at around 834 ppm to the presence of additional Mn^3+^ ions in the structure.^43^ The LNMO-O spectrum is deconvoluted in Figure S2, clearly revealing that the majority of lithium in the structure is at the ordered site. It is noteworthy that, to the best of our knowledge, such elevated degree TM ordering has not been reported for LNMO spinel phases previously. In the case of a random distribution of Mn and Ni atoms around Li in a LNMO spinel structure, an increased number of Li environments and consequently a more complex NMR spectra are expected. This is obviously the case in the ssNMR spectrum of LNMO-D, depicted in Figure 2b where a broader distribution of signals is observed between 600 and 1200 ppm. The resonance previously described at 1010 ppm is also present in the spectrum of LNMO-D and is similarly assigned to the ideal stoichiometry prevalent in the LNMO-O, although it has an evidently lower relative intensity and a larger broadening due to the extensive cation disorder present in the LNMO-D case. The higher disorder of LNMO-D is furthermore reflected by the presence of mutual signals at 935, 834, and 736 ppm which are clearly more intense than in LNMO-O, suggesting an almost random coordination of Mn/Ni as well as an additional shoulder observed around 1088 ppm. Analogous to LNMO-O, the spectrum of the LNMO-D was deconvoluted and is depicted in Figure S2. The shifts observed, see Table S1 are in very good agreement with the values observed in previous works.^2,43^ Specifically, signals at relatively lower chemical shift (935, 834, and 736 ppm) are assigned to lithium sites coordinated by more than nine Mn^4+^ cations (and fewer Ni^2+^) as compared to the ideal ordered structure. According to the results obtained by Cabana et al., the features shifted ≈70–75 ppm from the ideal ordered signal (at 1010 ppm) are tentatively assigned to single Ni^2+^/Mn^4+^ substitutions (see the Supporting Information for more details). In this work, signals at lower ppm were also observed shifted around 100 ppm. These signals were tentatively assigned to Mn^3+^ in Mn-rich regions.^2^ The presence of Mn^3+^ cations in our disordered LNMO-D sample is in agreement with the observed Mn^3+/4+^ redox activity around 4.1 V vs Li^+^/Li, which is more pronounced in LNMO-D than in LNMO-O (see Figure S1b). Furthermore, Duncan et al. have observed that the NMR spectra of Ni-deficient LNMO spinel structures containing Mn^3+^ are characterized by signals at 940, 840, and 740 ppm.^43^ The reported values are in very good agreement with those obtained in this work (935, 834, and 736 ppm), which supports the Ni-deficient stoichiometry in these samples, also evinced by ICP-AES and NPD. The signal observed at 1088 ppm in LNMO-D is assigned to Li with a local Ni-rich environment.^2^ Interestingly, such a Ni-rich feature is not observed in LNMO-O, which substantiates the hypothesis that the additional features observed in the TM-ordered LNMO do not originate from random intersite mixing but are due to Mn excess stoichiometry. The relative populations of each of the sites described are estimated from the deconvolution of the signals shown in Figure S2 and are reported in Table S1. It is noteworthy that the diamagnetic contribution, centered around ≈0 ppm, represents <1% of the total peak area, which underlines the purity of the materials, being virtually free of lithium containing diamagnetic species (e.g., Li_2_O and Li_2_CO_3_).

**Figure 3:**
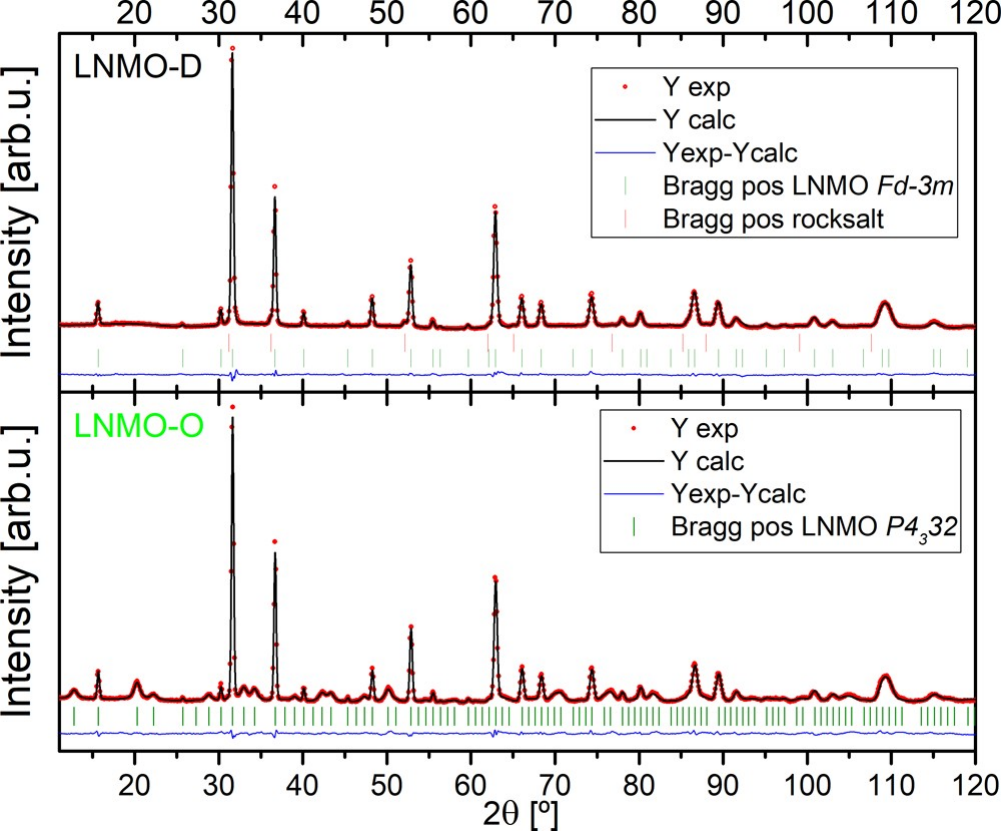
Rietveld refinement pattern of LNMO-D (top) and LNMO-O (bottom). A minority rock-salt phase was used besides the main spinel phase to refine the LNMO-D diffraction pattern. A single spinel phase including an antiphase model was used for LNMO-O pattern refinement.

Neutron powder diffraction (NPD) was performed to determine the crystal phase and more precisely characterize the TM ordering of both samples LNMO-D and LNMO-O. All sharp main diffraction reflections of the two samples coincide (see Figure 3) and can be attributed to spinel structure described in *Fd*3̅*m*. Thanks to the significantly different cross sections of Ni and Mn, NPD can reveal the TM ordering via the appearance of additional peaks linked to a superstructure. It is noteworthy that these superstructure features have a larger peak broadening than those attributed to the main reflections of the spinel phase, which has been previously observed by other groups.^11,47^ As proposed in the literature, the reduced symmetry cell of the TM-ordered LNMO phase can be indexed in the *P*4_3_32 space group.

The NPD pattern of the LNMO-D materials was refined by using Rietveld’s method against the *Fd*3̅*m* structural model of the disordered spinel (Figure 1a); a tiny rock-salt-type impurity (≈1%) was evinced to fully fit the pattern. In turn, LNMO-O’s NPD pattern was perfectly refined with a single phase of the *P*4_3_32 TM-ordered spinel phase by using an antiphase model to account for the anisotropic broadening of the superstructure peaks, as previously proposed.^11^ The Rietveld refinements of the NPD patterns of LNMO-D and LNMO-O are shown in Figure 3, and selected results are compared in Table 1. A more comprehensive description of the Rietveld refinements strategy as well as the final refined parameters for both materials are given in Table S2 in the supporting information. The Rietveld analysis reveals cubic lattice parameter of ≈8.18 Å for both samples, which is about 0.02  Å larger than those previously reported for coarse and stoichiometric LNMO,^5^ suggesting a lattice expansion due to Mn^3+^ presence as result of Ni-deficient stoichiometry. Moreover, the lattice constant of LNMO-O is slightly smaller than that of LNMO-D. This is in line with previous findings and has been attributed to a spontaneous spatial optimization of M–O bonds for ordered TM.^15^

**Table 1:** Selected Results of the Rietveld Refinements of the NPD Data

material	space group	lattice const [Å]	ϕ crystal size [nm]	phase cont [%]	χ^2^
LNMO-D	*Fd*3̅*m*	8.1862(4)	79	99.0(6)	50.3
LNMO-O	*P*4_3_32	8.1833(5)	124/6^a^	100	47.6

a Corresponds to antiphase domain size.

The size of the crystalline domains is around 100 nm for both materials with smaller domains for the LNMO-D. This is clearly smaller than the primary polygonal particles observed in SEM (see Figure S1), confirming their polycrystalline nature. Besides the crystalline domain size, Rietveld refinement also allows to estimate the size of antiphase domains for the LNMO-O sample, which is much smaller (≈6 nm) than the crystalline domain size and in accordance with the observed differences in peak width in the NPD pattern (see Figure 3, bottom). The refinement of the TM sites’ occupancies reveals a Mn/Ni ratio >3 for LNMO-D and LNMO-O, which confirms their Ni-deficient stoichiometry in agreement with findings of the ICP-AES (*vide supra*). For LNMO-O, the site occupancies reveal a highly ordered structure with Ni exclusively positioned in 4b and Mn predominantly in 12d sites; only a slight share of Mn is found in the 4b site to accommodate the Mn excess. This high degree of TM ordering is reflected by the prevalence of a single Li environment for LNMO-O as evinced by ^7^Li NMR (see Figure 2b). The appearance of the minority features observed in LNMO-O can hence be attributed to the presence of the Mn excess located at the 4b Ni site.

The above employed material characterization techniques have highlighted that the two LNMO samples investigated in this study are very similar in terms of phase purity, crystal structure, stoichiometry, and morphology and that the main difference lies in the degree of TM ordering. In this regard it is noteworthy that the LNMO-O sample has the highest degree of TM ordering so far reported in the literature.

### 3.2 *Operando* X-ray Absorption Spectroscopy

**Figure 4:**
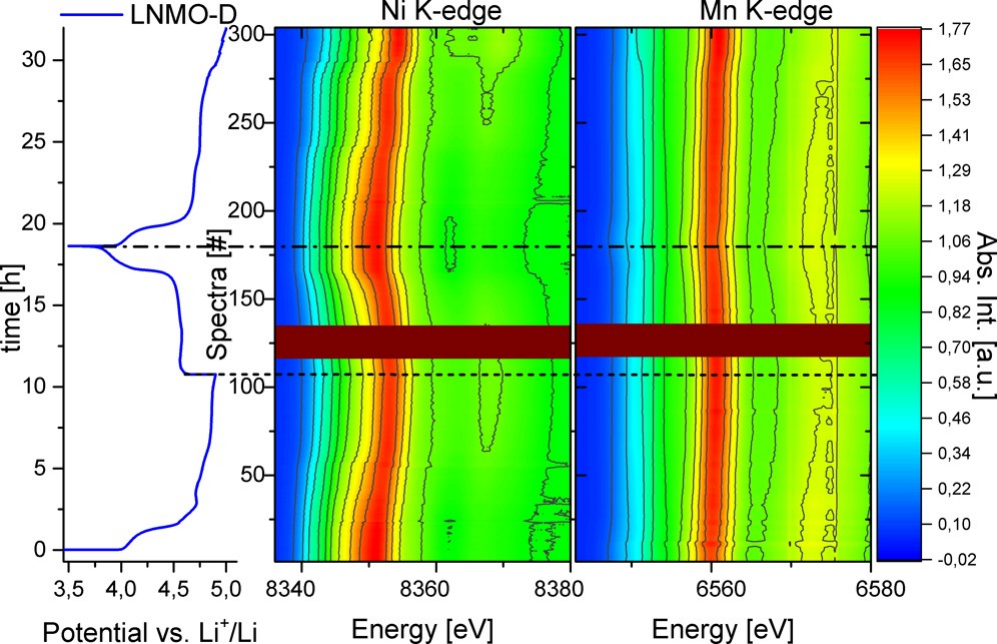
Evolution of XAS on Ni and Mn K-edge during 1.5 electrochemical cycles vs Li of LNMO-D. No spectra were acquired for the dark red region around spectra #125 due to a beam loss.

The evolution of *operando* absorption spectra during 1.5 electrochemical cycles of LNMO-D is depicted in Figure 4 along with the corresponding electrochemical cycling curve which reveals a short plateau with an onset at 4.0 V vs Li^+^/Li and an extended slight ascending slope followed by a plateau centered around 4.8 V vs Li^+^/Li, in agreement with cycling curve obtained under laboratory conditions (Figure S1b). The changes in the position and intensity of both TM absorption K-edges are clearly visible, which reflects their redox activity and local structural modification during the redox process. In this regard, it is salient that changes for the Ni K-edge are much more pronounced than for Mn. This suggests that Ni is the main redox-active TM, while the majority of Mn absorbers do not undergo significant changes. The contour plot reveals an onset of Ni spectral changes after spectra #25 corresponding to ≈4.7 vs Li^+^/Li, while for Mn spectral changes are observed prior to that. This points toward the previously reported attribution of the first plateau starting at 4.1 V vs Li^+^/Li to the Mn^3+/4+^ redox couple.^19^ Interestingly, a small but gradual change can be observed for the Mn absorption spectra beyond the region attributed to Mn redox. The possible implication of this will be discussed in more detail at a later stage.

The changes observed in the first charge (delithiation) up to 4.9 V vs Li^+^/Li, which extends to spectra #105, are reversed upon discharge (lithiation), resulting in similar spectral features after one complete cycle (#180) as the pristine state (#1). This spectral congruence is depicted in Figure S3 and underlines the reversibility of the redox reaction. Their overlap with NiO reference spectra confirms the prevalence of Ni^2+^ at these states of charge (SOC). In the second charge the cutoff voltage was raised to 5 V vs Li^+^/Li to ensure the complete oxidation of the TM. It is noteworthy that only minor changes in XAS are observed beyond spectra #290, suggesting that the observed plateau at 4.95 V can be predominantly attributed to parasitic oxidation reaction, as previously reported.^48^ With regard to the cell potential, it should be noted that an overpotential of ∼0.1 V was observed, which could be due to the high areal loading of the electrode to satisfy absorption intensity needs as well as to the specificity of the experimental conditions for the *operando* measurements.

The evolution of the TM-ordered phase LNMO-O, presented in Figure S4, reveals a similar picture as for LNMO-D. Within the first 25 spectra only Mn K-edge changes are observed, followed by a strong shift and intensity changes of the Ni K-edge. The changes observed in charge (delithiation) extending to spectra #141 are reversed during discharge up to spectra #218. Because of a cell failure, the second charge reaction was incomplete with only minor spectral changes observed after spectra #275.

**Figure 5:**
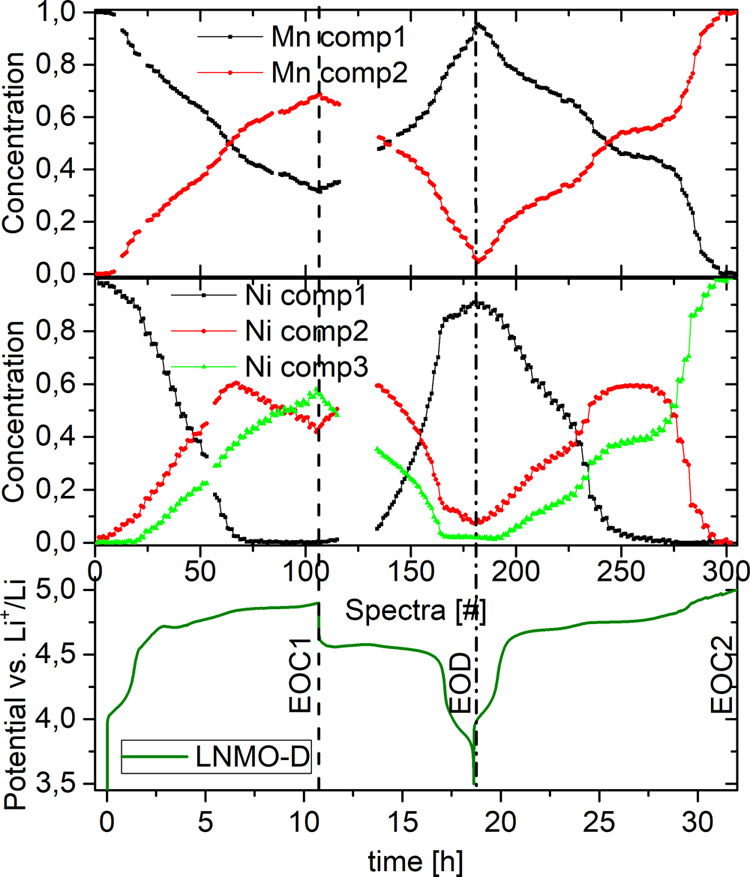
Concentration profile for Mn (upper) and Ni (lower) components upon 1.5 electrochemical cycles vs Li^+^/Li for LNMO=D. Gap in the concentration profile around spectra #125 is due to a beam loss. Vertical dashed and dashed-dotted lines indicate EOC1 and EOD, respectively.

To extract all relevant quantitative information in an unbiased and elegant way, a chemometric approach (PCA and MCR-ALS) was applied to the complete data sets comprising more than 1200 absorption spectra. Principal component analysis (PCA) indicates that the Ni K-edge data set is composed of three independent components, while for Mn two components are found for both LNMO types. The XANES region of these pure MCR-ALS spectral components are shown in Figure S6. The corresponding concentration profile of these components is shown in Figure 5 for LNMO-D.

At open-circuit voltage (spectra < #4) a single component is present for both Ni and Mn which can be identified as the pristine component. As a charge current is applied, the concentration of these pristine components gradually declines and component 2 arises. For Mn, this second component reaches its maximum at spectra #105, which coincides with the end of first charge (EOC1) at 4.9 V vs Li^+^/Li. No other components are observed for Mn during the charge/discharge reaction. Component 2 of Ni peaks at spectra #67, which corresponds to roughly 60% of charge capacity. This suggests that it represents a transient species in the charge reaction. The maximum of this transient species coincides with the onset of the second step of the extended plateau in the electrochemical cycling curve as well as with the complete depletion of the pristine component. Hence, beyond spectra #67 a mere transformation from Ni component 2 to component 3 can be observed upon continuous charge. Component 3 which starts to emerge at spectra #20 reaches its maximum at spectra #105, well in line with EOC1. At this point the Ni components 2 and 3 make up 40% and 60% and the Mn components 2 and 1 70% and 30%, respectively. This large remnant of Ni transient species at the EOC1 suggests that the charge reaction was incomplete at this point. During discharge, the reaction is inverted. First, Ni component 3 is transformed to component 2. Once it has reached its maximum, the pristine component arises again peaking at spectra #180, well in line with the end of discharge (EOD). It should be noted that only 90% of pristine component intensity is recovered after one complete cycle, which reflects a certain degree of irreversibility of the structural and chemical transformations occurring during the first complete delithiation–lithiation cycle. For the second charge, initially a similar trend is observed as during first charge. Instead of a sharp peak for Mn component 2 a plateau is observed around spectra #260 and 60% intensity. After this, a steep increase in Mn component 2 intensity is observed up to spectra #290, at which the concentration reaches 100%. Analogous to first charge, we observe for Ni concentrations that the transient component 2 reaches its maximum at around 60%, coinciding with the depletion of pristine component 1. Similarly to Mn, this maximum is not as sharp as in the initial charge. The fact that all TM concentrations stagnate around spectra #260, while at the same time the electrochemical curve continues to plateau, could reflect an inhomogeneous propagation of the reaction front or parasitic oxidation reactions. Upon further lithiation, a much faster transformation from Mn component 1 to component 2 and Ni component 2 to Ni component 3 is observed than in the first charge. Contrary to the first charge, Mn component 2 and Ni component 3 reach 100% at spectra #295, corresponding to the end of second charge (EOC2). This suggests a more complete charge reaction, which can be explained by the higher cutoff voltage in the second charge. Beyond spectra #295, corresponding to voltage >4.95 V vs Li^+^/Li, no changes in the K-edge are observed which infers that the ongoing charge reaction is due to parasitic reaction that do not involve the TM such as electrolyte decomposition.

Similarly, the concentration profile of the LNMO-O material can be examined which is depicted in Figure S6. The concentration profiles for Ni and Mn reveal a comparable global evolution as for LNMO-D. One noticeable difference is the fact that at EOD the Ni spectrum is entirely composed of component 3 while in LNMO-D a mix of components 2 and 3 is prevalent. This can be explained by the fact that a higher cutoff voltage of 5 V vs Li^+^/Li was applied already in the first charge for LNMO-O, and therefore a more complete state of charge was achieved. Unfortunately, because of a cycling issue, only the first part of the second charge up to spectra #250 is accurately captured for the LNMO-O. Beyond this point the charge curve deviates from the expected behavior, and spectral changes are small and have an elevated noise level.

**Figure 6:**
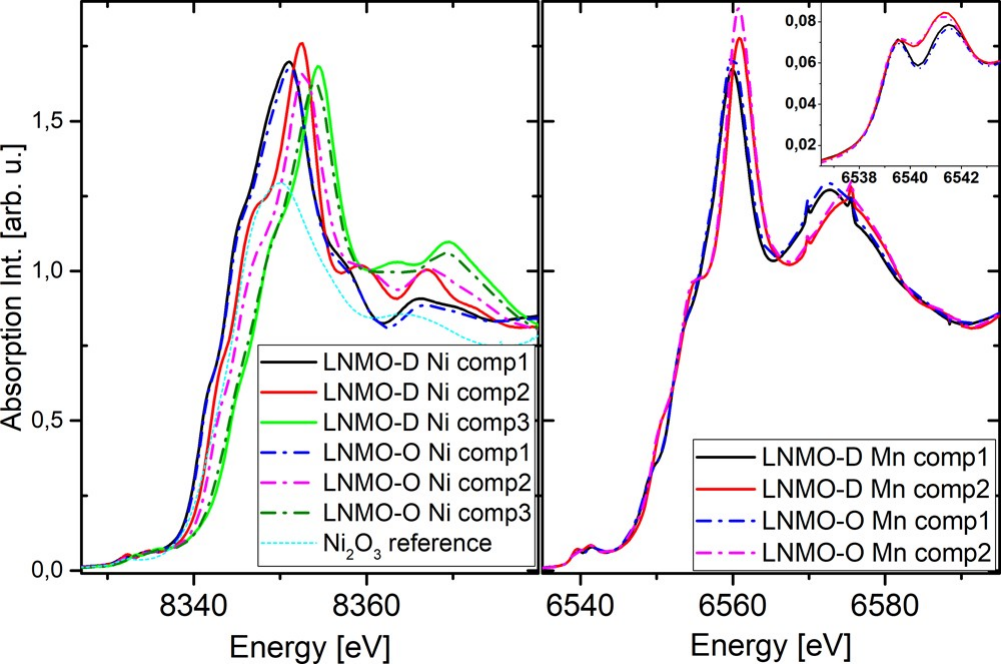
XANES K-edge region of MCR-ALS derived pure spectral components for Ni (left) and Mn (right). Spectral components for disordered phase LNMO-D are marked in solid lines and ordered LNMO-O in dash-dotted lines. A Ni_2_O_3_ reference spectra is traced with a dashed light blue line. The inset depicts the Mn pre-edge region.

The XANES of pure spectral components of LNMO-D and LNMO-O are presented in Figure 6. While both Mn components and Ni components 1 and 3 of the two materials coincide in terms of onset, shape, and white line position, there are slight differences for Ni component 2. The deviation in shape of the XANES suggests a difference in terms of coordination and/or symmetry of the Ni absorber in these components, which will be further investigated in the EXAFS analysis (*vide infra*). It is noteworthy that the edge position of the Ni component 2 is close to that of reference Ni_2_O_3_ spectra, suggesting a prevalence of the Ni(+III) oxidation state. The edge position of Ni component 2 of the LNMO-O is upshifted by 0.2 eV compared to LNMO-D, suggesting a slightly more oxidized state of Ni. Nevertheless, this energy difference corresponds to a mere 6% of total edge shift observed between components 1 and 3. Despite these minor deviations, the concordance in number of principal components, their spectral similarity, and their corresponding concentration profile suggest that both materials have similar reactant components and reaction pathways. Indeed, we observe that in both materials the Ni redox reaction occurs via formation of a transient species component 2. Interestingly, the three Ni components 1–3 coexist throughout a large part of the charge and discharge reactions for both LNMO-D and LNMO-O, however, with deviating dynamic which will be further discussed in section 4.

The continuous changes of the Mn K-edge throughout the entire electrochemical cycling curve which contradicts the expected redox activity limited around 4.1 V vs Li^+^/Li deserve a closer examination. First, it should be noted that Mn K-edge is complex since edge shifts are not always directly proportional to its oxidation state. Furthermore, it should be pointed out that the main K-edge is not an isolated signature of a single property, such as oxidation state, but is the result of a conglomerate of effects. The fact that only a small portion of the total Mn absorbers present in the LNMO are expected to be redox-active, further complicating the XANES analysis, as the spectral changes are “diluted”. To obtain a more reliable picture of the oxidation state changes, we have examined the Mn pre-edge which is located about 20 eV before the main edge (6538–6544 eV). Unlike the main edge which arises from the 1s → 4p transition, these forbidden 1s → 3d transitions directly probe the redox relevant d orbital. Hence, the pre-edge features are much less affected by changes in the medium- and long-range environment than the main-edge region.^49,50^ The pre-edge evolution of Mn K-edge of LNMO-D during electrochemical cycling is depicted in Figure S7 (right). Because of d-orbital splitting in octahedral environment, it is composed of two peaks, namely t_2g_ and e_g_ at 6539.5 and 6541.5 eV, respectively. A strong increase of the e_g_ pre-edge feature intensity within the first 50 spectra of the charge reaction can be observed. This is interpreted as the emptying of the 3d e_g_ orbitals (from d4 to d3) due to Mn^3+^ to Mn^4+^ oxidation in an octahedrally coordinated high-spin electronic configuration. For the t_2g_ peak a mere broadening can be observed which could be attributed to site distortion, linked to Jahn–Teller effect prone Mn^3+^. This trend is reversed within the last 30 spectra of the discharge, underlining the reversibility of these electronic and structural changes. To quantify these spectral changes, we performed MCR-ALS analogous to above presented results but limited to the Mn pre-edge K-edge energy range data set. The resulting concentration evolution of the two components (full markers) are shown in Figure S7 (left) and compared to the concentration profile obtained based on full edge spectra (hollow markers). The comparison of the two concentration profiles reveals a much sharper rise of the component 2 for pre-edge based data set than for full edge energy range data. Indeed, for the pre-edge based data the main changes occur within the first 50 spectra followed by a plateau for almost 100 spectra before sharply declining after spectra #150. This indicates that the main spectral changes in the pre-edge region are occurring at the beginning of the charge and end of the discharge reaction, at operating voltages well below the Ni plateau of 4.7 V in line with Mn’s expected redox activity.

Interestingly, we observe a similar trend for the Mn K-edge pre-edge features of the LNMO-O phase. This is exemplified by the similar spectral components in the Mn K-edge pre-edge range (Figure 6, inset). This underlines that Mn redox activity is not exclusive to the TM disordered materials but can also occur in rock-salt-free, highly TM ordered materials.

**Figure 7:**
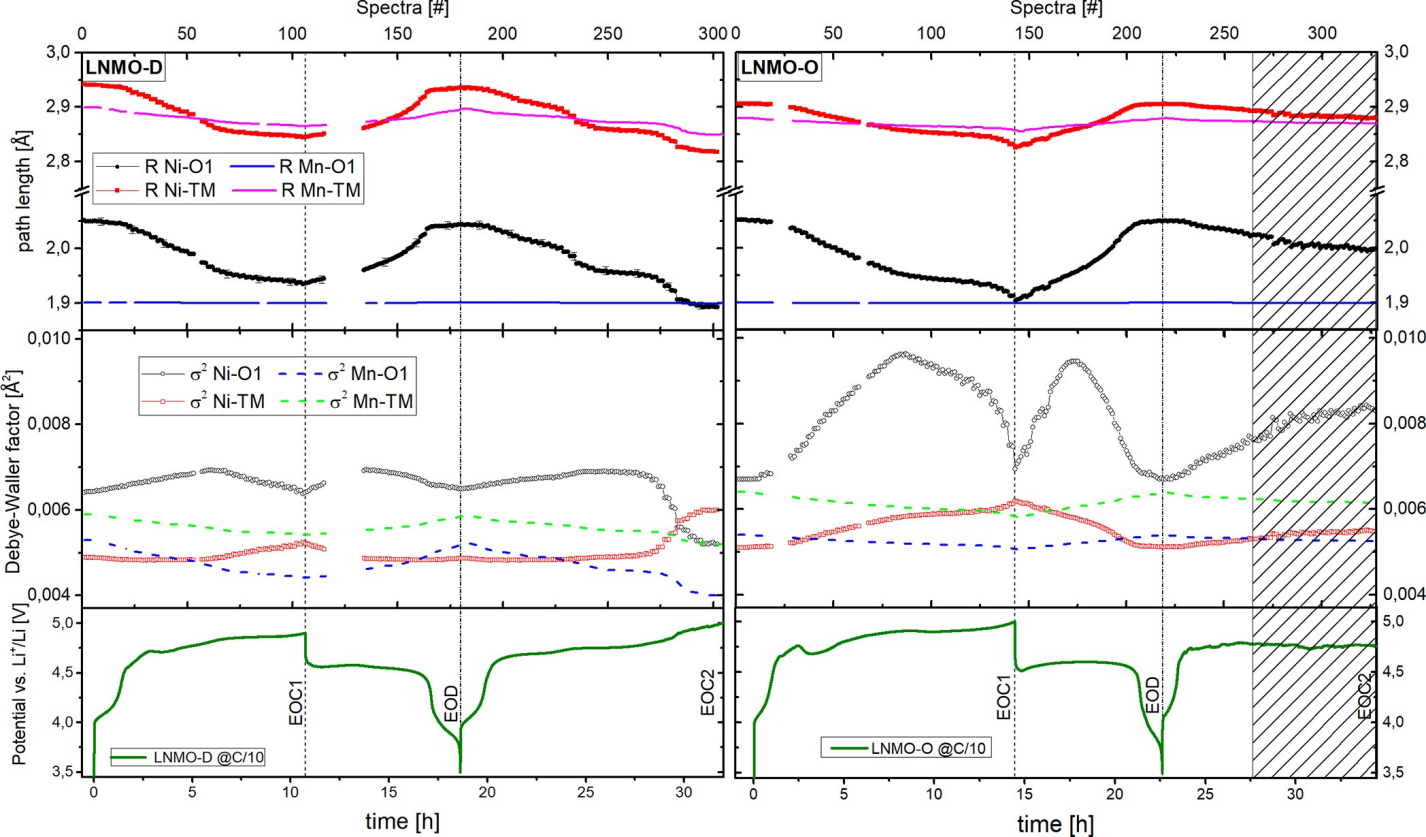
Evolution of path length (top) and Debye–Waller factor (middle) of two closest Ni–next neighbor shells (Ni–O1 and Ni–TM) upon electrochemical cycling of LNMO-D (left) and LNMO-O (right). The solid and dashed lines without markers depict the path length and Debye–Waller factor of Mn shells (Mn–O1 and Mn–TM). Error bars fall within the width of markers and have therefore been omitted. The gaps are due to beam loss. Vertical dashed and dashed-dotted lines indicate EOC1 and EOD, respectively. The shaded area indicates nonconformity of cell behavior during the second charge of LNMO-O.

To get quantitative information about local structure of the redox active TM, the EXAFS spectra of the identified MCR-ALS components were fitted. The parameters of the fitting are presented in Tables S3 and S4 for LNMO-D and LNMO-O, respectively. By coupling these fitting parameters with the concentration profile derived from MCR-ALS, we can reconstruct the evolution of the local structure upon electrochemical cycling. The evolution of the interatomic distance (path length, *R*) for the two closest Ni shells, namely Ni–O1 and Ni–TM, which make up the lion’s share of scattering intensity, along with corresponding Debye–Waller factor (σ^2^) are depicted for LNMO-D and LNMO-O on left and right side in Figure 7, respectively.

For both Ni bond distances (Ni–O1 and Ni–TM, black and red solid markers in Figure 7 (top), respectively) of the LNMO-D, we observe a mutual trend of gradual decrease upon charge and increase upon discharge. This is in good agreement with previous reports and has been attributed primarily to the shrinkage of ionic radii of the TM upon oxidation.^28,36,46^ This observation suggests that the Ni–O_6_ octahedra as well as the further outlying Ni–TM shell are equally affected by the reversible removal and reinsertion of Li. For Mn, on the other hand, no significant change in the average interatomic distance of the Mn–O_6_ octahedra is observed (blue line without markers in Figure 7, top), while the Mn–TM shell (magenta line without markers) follows a similar trend as the Ni–TM, although with an attenuated amplitude. According to Bathia et al., this discrepancy can be explained due to the fact that the Mn^3+^ to Mn^4+^ transition does not noticeably affect the Mn–O bond length.^28^ One of the root causes for this, is a smaller change in ionic radii upon oxidation of Mn compared to Ni. While a mere 5 pm reduction is reported for Mn^3+^ to Mn^4+^, the Ni^2+^ to Ni^4+^ oxidation involves a 21 pm reduction in ionic radius.^51^

For the Debye–Waller factor (σ^2^), which represents the variation in the distribution of bond lengths, we observe a more intricate picture. For the Ni–O_6_ octahedra (black solid markers in Figure 7 (middle)), a gradual increase up to spectra #65 can be observed. Upon further charge it declines to starting value. This finding is well in line with a previous EXAFS study, reporting a peak value of σ^2^ for the half-charged state at 4.7 V.^36^ Upon discharge an analogous evolution is observed. This trend concurs well with the rise and fall of intermediate Ni component 2, which implies that the formation of this transient Ni^3+^ phase involves a broader distribution of bond lengths in the Ni–O octahedra. The σ^2^ of Ni–TM shell shows a local maximum at spectra #110, which corresponds to the end of charge reaction (EOC1). This trend is reversed upon discharge. For the σ^2^ of the two Mn shells (dashed blue and green line without markers) a mutual trend of gradual decrease upon charge and increase upon discharge is found. A special note should be made concerning the region beyond spectra #275, which correspond to the second charge beyond 4.8 V. For this region a shortening in interatomic distance as well as change in σ^2^ beyond those in the first charge are observed. Regardless of these trends, it should be noted that the absolute changes in interatomic distance as well as Debye–Waller factor are comparatively small and underline the preservation of (local) structural integrity of the LNMO host structure upon reversible intercalation of lithium.

For LNMO-O (see Figure 7, right) we find many similarities to LNMO-D in terms of starting values for the pristine material as well as general trends for the bond length and Debye–Waller factor evolution upon delithiation and lithiation. The average bond distance Ni–TM of pristine LNMO-O is slightly shorter than that of pristine LNMO-D, which could be linked to the slightly smaller lattice parameter for the *P*4_3_32 phase (see Table 1). Nevertheless, comparable changes in bond distances are observed as for LNMO-D. The most salient difference is the much stronger change of σ^2^ upon cycling in LNMO-O compared to LNMO-D. While starting values of σ^2^ for both Ni shells of pristine LNMO-D and LNMO-O are nearly the same, a much more pronounced increase is observed upon charge for LNMO-O, particularly for the Ni–O1 octahedral bond, which will be further discussed in the following section. The shaded area in Figure 7 (right) marks a region of electrochemical cycling issues in which electrochemical cycling curve deviates from expected signature and thereby compromises the XAS results.

## 4 Discussion

Our findings underline that the redox reaction of TM ordered and disordered LNMO is primarily based on the Ni^2+/4+^ redox couple. The here applied unbiased chemometric approach reveals the existence of three linearly independent Ni components, which is well in agreement with previously proposed three consecutive cubic phases Li_1_Ni_0.5_Mn_1.5_O_4_, Li_0.5_Ni_0.5_Mn_1.5_O_4_, and Li_0_Ni_0.5_Mn_1.5_O_4_.^23,52^ Furthermore, we have evinced the formation of a transient phase (Ni component 2) which culminates at about halfway through the Ni plateau. Rendering the formation of such transient/intermediate phases visible, out of the vast spectroscopic data set, is one of the key assets of the chemometric approach.^40^ This transient component was assigned to the Ni^3+^ (Li_0.5_Ni_0.5_Mn_1.5_O_4_) phase. In this regard we validate the previously proposed single electron transfer mechanism from Ni^2+^ → Ni^3+^ → Ni^4+^ by Qiao and co-workers based on selected *ex situ* soft XAS^35^ as well as via *ex situ* Raman spectroscopy.^28^ Ni component 3, which represents the end of charge (EOC) state, can be attributed to the Ni^4+^ phase. It should be noted that the latter starts to emerge already at an early stage of the charge reaction, yielding to the effective coexistence of Ni^2+^ and Ni^4+^ phase which alludes to a nonuniform redox reaction propagation. Interestingly, while this coexistence is found in both LNMO-D and LNMO-O, their dynamics are clearly distinct. In the LNMO-D, an earlier and steeper rise of component 3 is observed than in LNMO-O, resulting in an intersection of components 1 and 3 at ≈33% intensity, while in LNMO-O they intersect at a mere 5%. From this, we can assume that the lithiation in LNMO-O proceeds more homogeneously than in the LNMO-D sample. To the best of our knowledge, only few previous *in situ* or *operando* studies have depicted the phase evolution for the cycling mechanism of LNMO.^23,28,30^ Unfortunately, because of limited reaction step resolution (measurement points) and accuracy, those studies provided only a simplified schematic of subsequent Ni^2+^ → Ni^3+^ and Ni^3+^ → Ni^4+^ reactions and fail to evince the overlap of the three Ni phases. In fact, in their comparative study of XAS and XRD Arai et al. show a three Ni phase coexistence only for XAS-based data analysis while this is not evinced in their XRD data. This underlines that the here applied *operando* XAS in combination with chemometric data analysis provides new additional insights into the dynamics of LNMO’s cycling mechanism.

Besides the primary redox-active species Ni, a minority redox activity of Mn has also been evinced. We have recalled that shape and position of Mn main K-edge are strongly affected by the geometry, coordination, and oxidation state of its next neighbors. This interdependence compromises its meaningfulness for monitoring redox activity of the Mn absorber. Restricting the view to the pre-edge energy range can partially suppress these side effects and hence allow a more reliable view of the Mn^3+/4+^ redox couple. Additionally, we highlight the problem of oxidative stability as our measurements reveal that charge reaction at elevated potential is at least partially driven by reactions that do not involve TM redox and are hence undesired, such as electrolyte decomposition.

A comparison of LNMO-D and LNMO-O from a spectroscopic viewpoint reveals that electronic and local structure undergo similar evolution as their corresponding concentration profile and spectral components are resembling. This infers an analogous sequence of redox reaction involving similar reactants and intermediates, despite the proposed different reaction mechanisms (biphasic vs solid solution).^4,10^ This is hardly surprising as our chemometric analysis is based on the average oxidation state of the TM absorber (XANES) which is blind to the proposed phase difference. For evincing such a difference in phase mechanism, an EXAFS analysis is required which will be further discussed below. Interestingly, both LNMO-D and LNMO-O show reversible Mn redox contribution, which suggests the existence of Mn^3+^ in both materials. Its presence is hence not exclusively induced by additional heat treatment which is performed to transform the ordered *P*4_3_32 spinel phase to the disordered *Fd*3̅*m* one, as previously claimed.^13^ In this regard, our findings are well in line with a previous XAS-based study showing Mn redox activity in TM-ordered LNMO.^36^

The evolution of the bond length obtained from EXAFS analysis allows an indirect measure of the bond covalency, reflecting the TM oxidation state. Because bond lengths, unlike K-edge position, are less affected by coordination or symmetry, it can be regarded as a more conclusive measure.^36^ Our findings show a similar trend of bond length evolution for LNMO-D and LNMO-O, which suggests that the two closest next-neighbor shells are equally affected by lithium deinsertion and insertion in the *Fd*3̅*m* and *P*4_3_32 phases. At first glance our local structural findings seem to be in contradiction to those from previous ^7^Li and ^6^Li NMR studies, which reveal distinct behavior for TM-ordered and disordered spinel phases upon electrochemical cycling.^30^ However, these discrepancies can be explained by their different probing perspective. While NMR probes the local environment of mobile Li species, the here presented XAS probes the local structure of the redox-active TM–oxide scaffold structure with Li being X-ray transparent. The results can hence not be directly compared.

A noticeable difference in our local structural analysis between the ordered and disordered phases lies in the evolution of the Debye–Waller factor, which, assuming that local temperature remains constant, can be attributed to the variance in bond length, whereas a higher σ^2^ value suggests a broader distribution of the bond length. We find that the formation of the transient Ni component 2 is accompanied by an increase in Debye–Waller for both LNMO phases. Such an increase could be linked to the Jahn–Teller induced anisotropic lattice changes of the Ni^3+^ (d7) phase or a phase coexistence. Interestingly, a much larger bond length distribution of the Ni–O octahedra is observed for the ordered phase than for the disordered phase. This can be understood in the context of the previously proposed two-phase vs solid–solution reaction mechanism for this first part of the charge reaction for the ordered *P*4_3_32 and disordered *Fd*3̅*m* spinels, respectively.^4,10,47^ In this regard, the increased σ^2^ of LNMO-O at the end of first Ni plateau reflects the coexistence of two phases with different Ni–O bond distances. Whether the phase coexistence is the sole reason for the increased Debye–Waller or there is also a stronger distortion of the local environment in the TM ordered spinel phase (*P*4_3_32) cannot be answered with certainty at this point. Nevertheless, the magnitude of lattice distortion contribution on the Debye–Waller can be estimated by the changes on σ^2^ observed for LNMO-D, for which a solid solution mechanism has been proposed. This example illustrates that by carrying out a thorough EXAFS analysis of the local structure of all the participating reactants, the differences in reaction mechanism of solid solution vs two phase mechanism can be indirectly revealed, which would not have been possible based on simple XANES analysis.

Summarizing, we can conclude that although the reaction pathway and participating species are similar for the LNMO-D (*Fd*3̅*m*) and LNMO-O (*P*4_3_32) phases, there are noticeable differences in terms of dynamic and local structure evolution. The lithiation propagation in LNMO-O is more homogeneous than in LNMO-D, and the formation of the transient Ni^3+^ (Li_0.5_Ni_0.5_Mn_1.5_O_4_) infers an increased local structural disorder in the LNMO-O. Because other material properties such as morphology, phase purity, and stoichiometry of the LNMO-O and LNMO-D samples studied here are very similar, we can conclude that these deviations can be attributed to the difference in TM ordering.

## 5 Conclusion

In this study, we compare the electrochemical redox mechanism vs Li of transition-metal ordered and disordered LNMO via dual-edge *operando* XAS, analyzed with a reliable and unbiased statistical approach. Our results reveal that in both materials a consecutive redox reaction of Mn and Ni occurs and that the redox reactivity is predominantly reliant on the Ni^2+/4+^ redox couple involving the formation of a transient Ni^3+^ phase (Li_0.5_Ni_0.5_Mn_1.5_O_4_), in accordance with previous results. Furthermore, the comparison of Mn K-edge main edge and pre-edge evolution highlights their different sensitivity to the interdependencies of coordination and oxidation state of the Mn transition-metal absorber center and its next neighbors. Our in-depth comparison of the reaction process and electronic and local structural evolution for both phases show strong similarities; nevertheless, noticeable differences are revealed. We evince that the reaction propagation of highly TM-ordered LNMO is more homogeneous than for the TM-disordered one. Furthermore, we highlight that the formation of transient Ni^3+^ species is linked to increased local structural disorder in the TM-ordered LNMO compared to TM-disordered LNMO. Last but not least, we have demonstrated that by coupling Rietveld refinement of NPD with Li NMR analysis results valuable insights into the degree and nature of the TM intersite mixing can be gained. We hope that these insights will foster the material development of LNMO spinel materials for meeting the challenging requirements of next-generation lithium ion batteries.

## Supporting Information Available

SEM micgrograph and electrochemical cycling curve; deconvoluted NMR spectra; NPD refinement parameters; contour plot of Ni and Mn K-edge spectra data set for LNMO-O; variance and evolution of PCA components for Ni K-edge data set; concentration profile of Mn and Ni K-edge spectra for LNMO-O; comparison of concentration profile of pure spectral components of Mn pre-edge and full edge data set and Mn pre-edge contour plot; fitted EXAFS spectra of pure components; EXAFS fitting parameters of LNMOD and LNMO-O (PDF)
