# Supplementary material for: Influence of Transition-Metal Order on the Reaction Mechanism of LNMO Cathode Spinel: An Operando X-ray Absorption Spectroscopy Study
Source: Chem Mater. 2022 Jul 6;34(14):6529–40. doi: 10.1021/acs.chemmater.2c01360 (PMC9332344; doi:10.1021/acs.chemmater.2c01360)
Supplement: Supplementary file 1 — cm2c01360_si_001.pdf [file cm2c01360_si_001.pdf]

Supporting Information: Influence of transition metal order on the reaction mechanism of LNMO cathode spinel: An *operando* x-ray absorption spectroscopy study

Marcus Fehse,[*,†] Naiara Etxebarria,[†] Laida Otaegui,[†] Marta Cabello,[†] Silvia Martín-Fuentes,[†] Maria Angeles Cabañero,[†] Iciar Monterrubio,[†,‡] Christian Fink Elkjær,[¶] Oscar Fabelo,[§] Nahom Asres Enkubari,[†] Juan Miguel López del Amo,[†] Montse Casas-Cabanas,[†,‖] and Marine Reynaud[†]

†*Center for Cooperative Research on Alternative Energies (CIC energiGUNE), Basque Research and Technology Alliance (BRTA), Alava Technology Park, Albert Einstein 48, 01510, Vitoria-Gasteiz, Spain*

‡*Inorganic chemistry Department, Science and Technology Faculty, Basque Country University (UPV/EHU), 48940 Leioa, Bilbao, Spain*

¶*Haldor Topsoe A/S, Haldor Topsøes Allé 1, 2800 Kgs. Lyngby, Denmark*

§*Institut Laue Langevin, 38042 Grenoble Cedex, France*

‖*Ikerbasque - Basque Foundation for Science, Maria Diaz de Haro 3, 48013 Bilbao, Spain*

E-mail: marcus.fehse@umontpellier.fr

NMR ^{7}Li NMR spectra of diamagnetic materials are dominated by the quadrupolar interaction and the homonuclear dipolar coupling, and some residual broadening often remains,

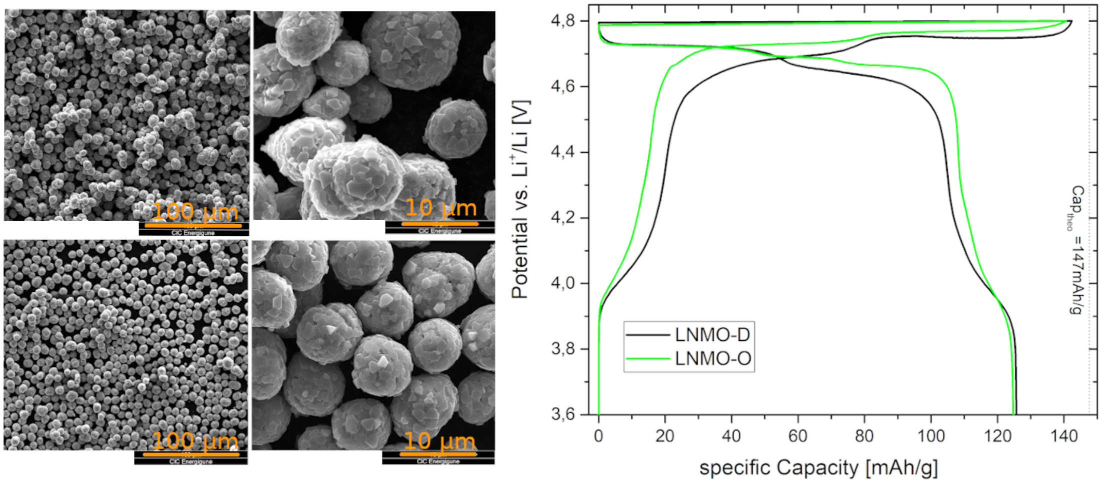

Figure S1: (a) SEM micrographs of pristine LNMO-D (top) and LNMO-O (bottom).(b) Electrochemical signature of LNMO-O and LNMO-D in coin cell half cells vs. Li^+/Li at C/20.

even under fast MAS conditions. In contrast to ^{7}Li, the much smaller quadrupolar moment and weaker homonuclear dipolar coupling of ^{6}Li allows for increased spectral resolution and fewer spinning sidebands. The electron-nuclear dipolar coupling is much smaller for ^{6}Li, due to its smaller gyromagnetic ratio, and thus the ^{6}Li spectra of these materials contain fewer spinning sidebands in the MAS spectra, which facilitates the spectral attribution.

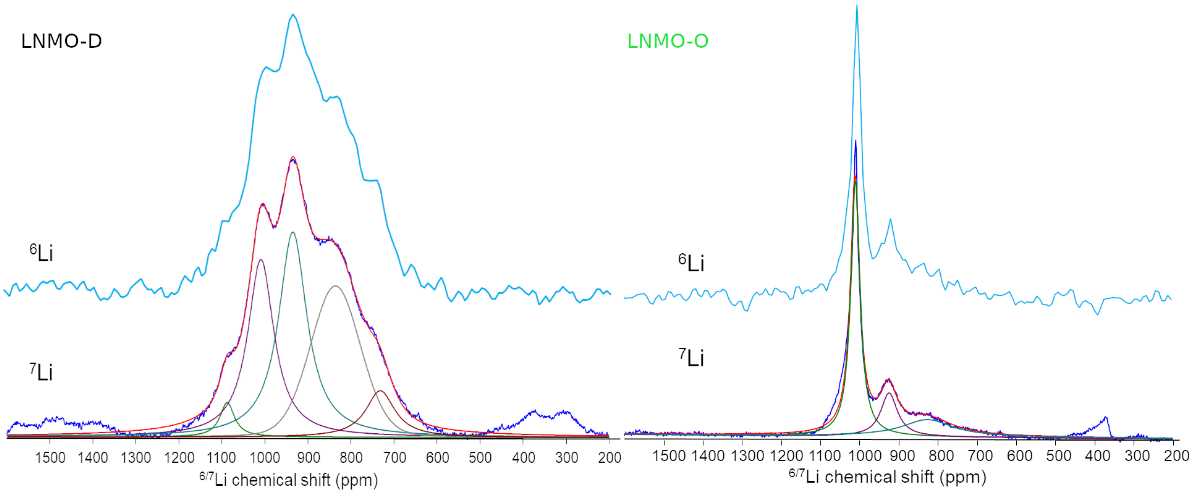

Figure S2: Deconvolution of the ^{7}Li MAS NMR spectrum of LNMO-D and LNMO-O on left and right, respectively. Dark blue: experimental data; red: additive theoretical spectrum and deconvoluted peaks. ^{6}Li MAS NMR stacked on the top (light blue).

Cabana et al. observed the trend that resonances at the lowest ppm was shifted by

approximately 100 ppm from the next higher frequency resonance, while the other signals were separated by approximately 70-75 ppm [S1]. They attribute this larger shift to the effect to the difference in oxidation state of Mn, assigning this environment to Li in vicinity of more Mn^{3+} ions. Interestingly, similar relative shifts are observed from the deconvolution in this work, see Tab. S1, although the absolute positions differ from those previously reported.

Table S1: Peak intensity of deconvoluted ^{7}Li NMR spectra for LNMO-D and LNMO-O.

**≈ ^{7}Li shift [ppm]**	LNMO-D [%]	LNMO-O [%]
1088	2.3	0
1010	23.6	59.9
935	33.2	16.6
834	36.1	23.5
736	4.7	0

A closer look at the deconvoluted spectra of LNMO-O Fig. S2(right), reveals slight deviations in intensity between the fit and the experimental data around 1010 ppm. These deviations reflect the need for additional peaks in the deconvolution, implying that the real structure is more complex than the simple model used here consisting of the exchange Ni/Mn coordination and the presence of Mn^{3+}. A tentative explanation for such upshifted features are locally Ni-rich regions with ideal stoichiometry but with different configuration on oxygen site such: O–Ni_2Mn configuration in a very local Ni-rich region (O-site). This configuration has been proposed before with *ab initio* simulations based on density functional theory study [S2].

NPD The NPD pattern of the sample LNMO-D was refined using Rietveld's methods, against the structural model of the TM-disordered spinel described in the $Fd\bar{3}m$ unit cell. The relative occupancy of Mn/Ni on the 16d site was refined, constraining the sum of contributions of the two metal for a full occupation of the site. Isotropic factors Biso were refined for all atoms. For this sample, a minor rock-salt-type impurity was detected. It was included in the refinement as a $Li_{0.4}Ni_{1.6}O_2$ phase (ICSD Collection Code #71422) in

Rietveld mode, but without refining the structural parameters except the cell parameters. This impurity was refined to account for 1(0.6)% of the crystalline phases present in the sample.

The NPD pattern of sample LNMO-O was refined in Rietveld mode against the superstructural model of the TM-ordered spinel described in the $P4_332$ space group. All reflections were fully fitted using this single phase; no crystalline impurity was detected in this sample. The anisotropic broadening of the superstructure peaks was perfectly refined using the antiphase model proposed in a previous publication [S3].

Table S2 presents the results of the Rietveld refinement of the NPD patterns of samples LNMO-D and LNMO-O. The 12d site was refined to be fully occupied by Mn, while the 4b site was evinced to accommodate the small excess of Mn atoms, as detected from ICP and NMR. Isotropic factors Biso were refined independently, except for those atoms occupying the same position, which were refined constrained to be equal.

XAS In this paragraph additional details on the XAS spectra, on the results of the PCA MCR-ALS analysis as well as on the EXAFS fitting are presented.

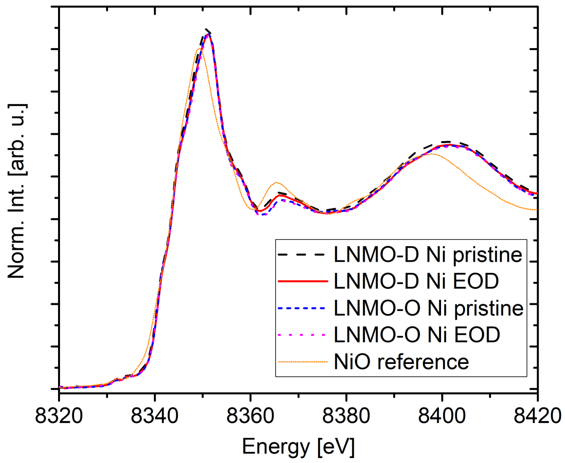

Figure S3: Experimental XANES spectra of Ni K-edge of LNMO-D and LNMO-O at pristine state and after one complete electrochemical cycle (EOD) vs. Li^+/Li. Short dotted orange thin line depicts XANES spectra of NiO reference.

Table S2: Additional Rietveld refinement parameters of the neutron powder diffraction data.

**Material**	LNMO-D		LNMO-O	
spacegroup	$Fd\bar{3}m$		$P4_332$	
Fractional coordinates	Mn/Ni x,y,z (16d)	0.5	Mn/Ni x (12d)	0.125
			Mn/Ni y (12d)	0.381(4)
			Mn/Ni z (12d)	0.870(4)
			Mn/Ni x,y,z (4b)	0.625
	Li x,y,z (8a)	0.125	Li x,y,z (8c)	0.005(4)
	O x,y,z (32e)	0.2629(2)	O1 x,y,z (8c)	0.3847(6)
			O2 x (24e)	0.1497(8)
			O2 y (24e)	0.8580(9)
			O2 z (24e)	0.1247(7)
Occupancies*	Mn/Ni (16d)	1.579(4)/0.416(4)	Mn (12d)	1.5
			Ni/Mn (4b)	0.454(3)/ 0.046(3)
Biso (Å^2)	Mn/Ni x,y,z (16d)	0.7(3)	Mn (12d)	0.5(2)
			Ni/Mn (4b)	0.7(2)
	Li x,y,z (8a)	1.3(4)	Li(8c)	1.2(4)
	O1 x,y,z (32e)	0.94(5)	O1(8c)	0.4(2)
			O2 (24e)	0.55(7)
Quality indicators	R_p	9.62		8.17
	R_{wp}	8.53		8.72
	R_e	1.21		1.26

* Sum of TM has been constrained

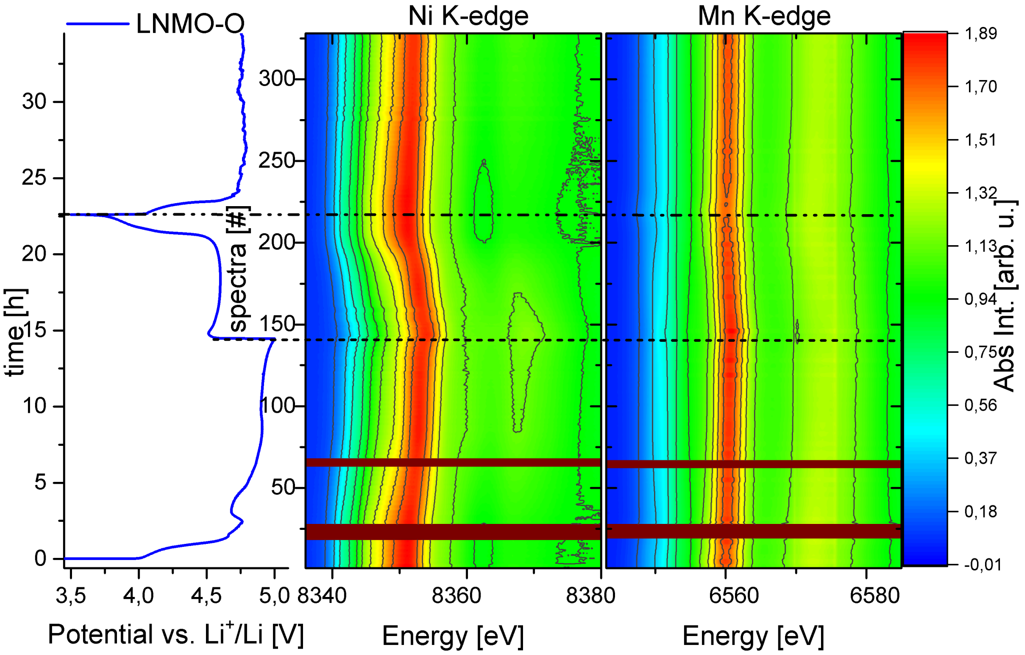

Figure S4: Evolution of XAS on Ni and Mn K-edge during 1.5 electrochemical cycles vs Li^+/Li. No spectra were acquired for the dark red coloured region around spectra #24 and #64 due to beamloss.

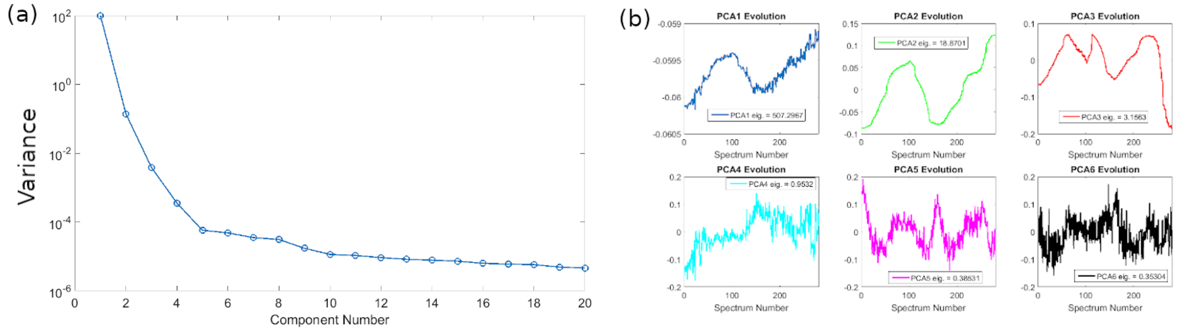

Figure S5: PCA derived (a) evolution of eigenvalue of components in order of decreasing variance and (b) evolution of first 6 components throughout the operando experiment.

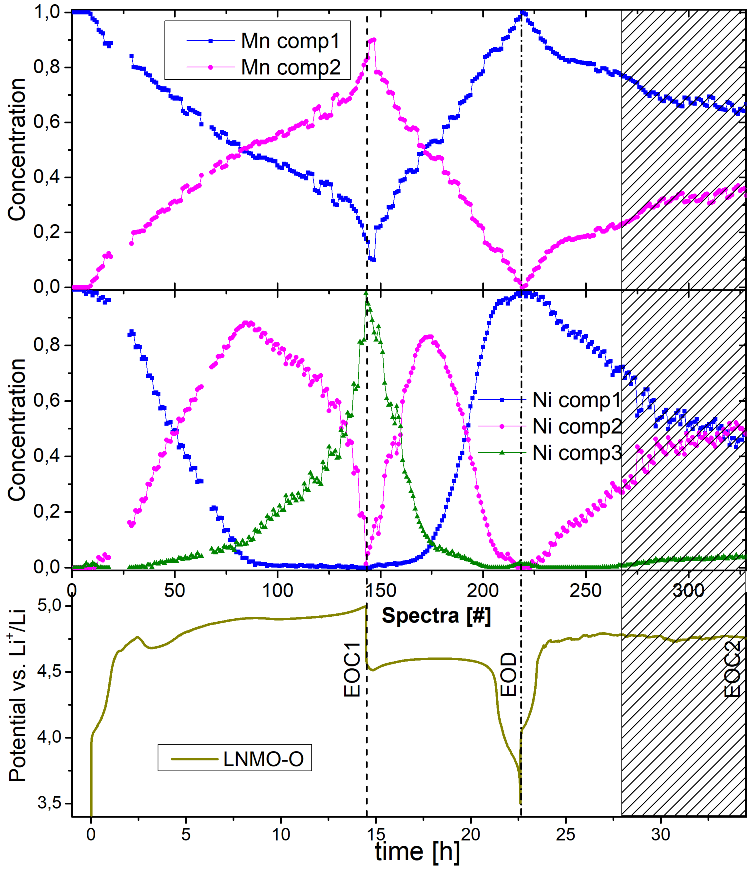

Figure S6: Concentration profile for Mn (upper) and Ni (lower) components upon 1.5 electrochemical cycles vs. Li^+/Li for LNMO-O. Vertical Gaps in the concentration profile around spectra #24 and #64 are due to beamloss. Vertical dashed and dashed-dotted lines indicate EOC1 and EOD, respectively.During second charge a cycling issue occurred which led to deviation from expected electrochemical signature beyond spectra #260 indicated by shaded area.

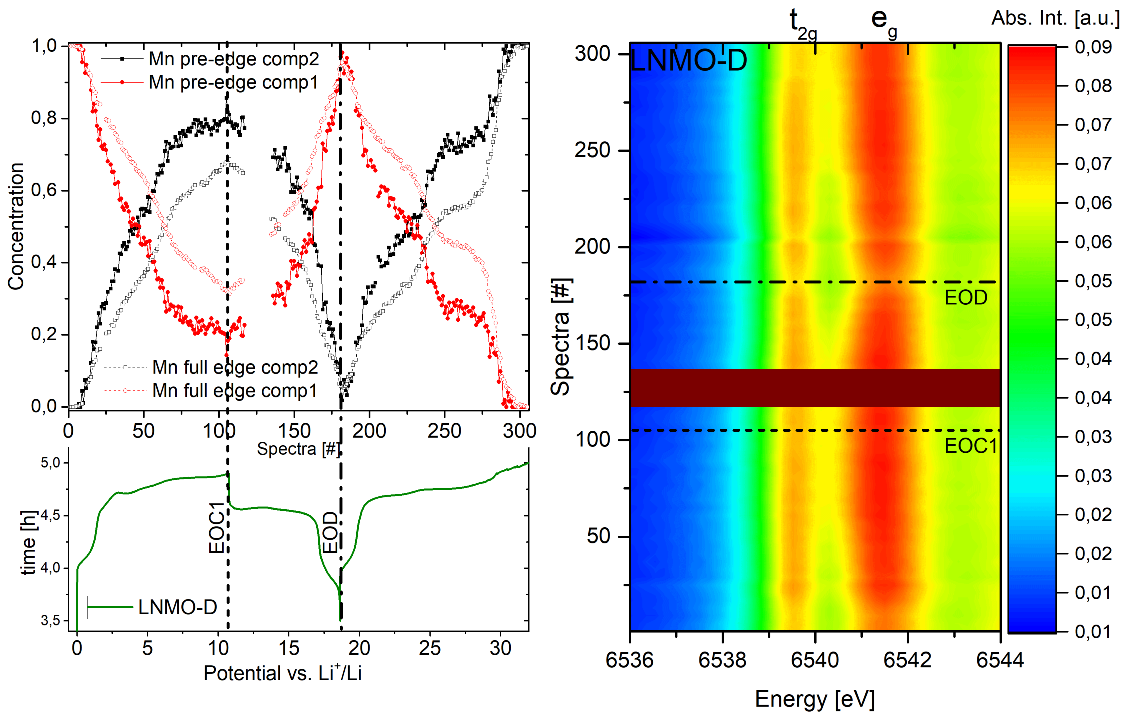

Figure S7: Left: MCR-ALS derived concentration profile for Mn components based on pre-edge (solid markers) and full edge region (hollow markers) upon 1.5 electrochemical cycles vs. Li^+/Li for LNMO-D. Gap in the concentration profile around spectra #124 are due to beamloss. Right: Contour plot of Mn K-edge pre-edge region during electrochemical cycling.

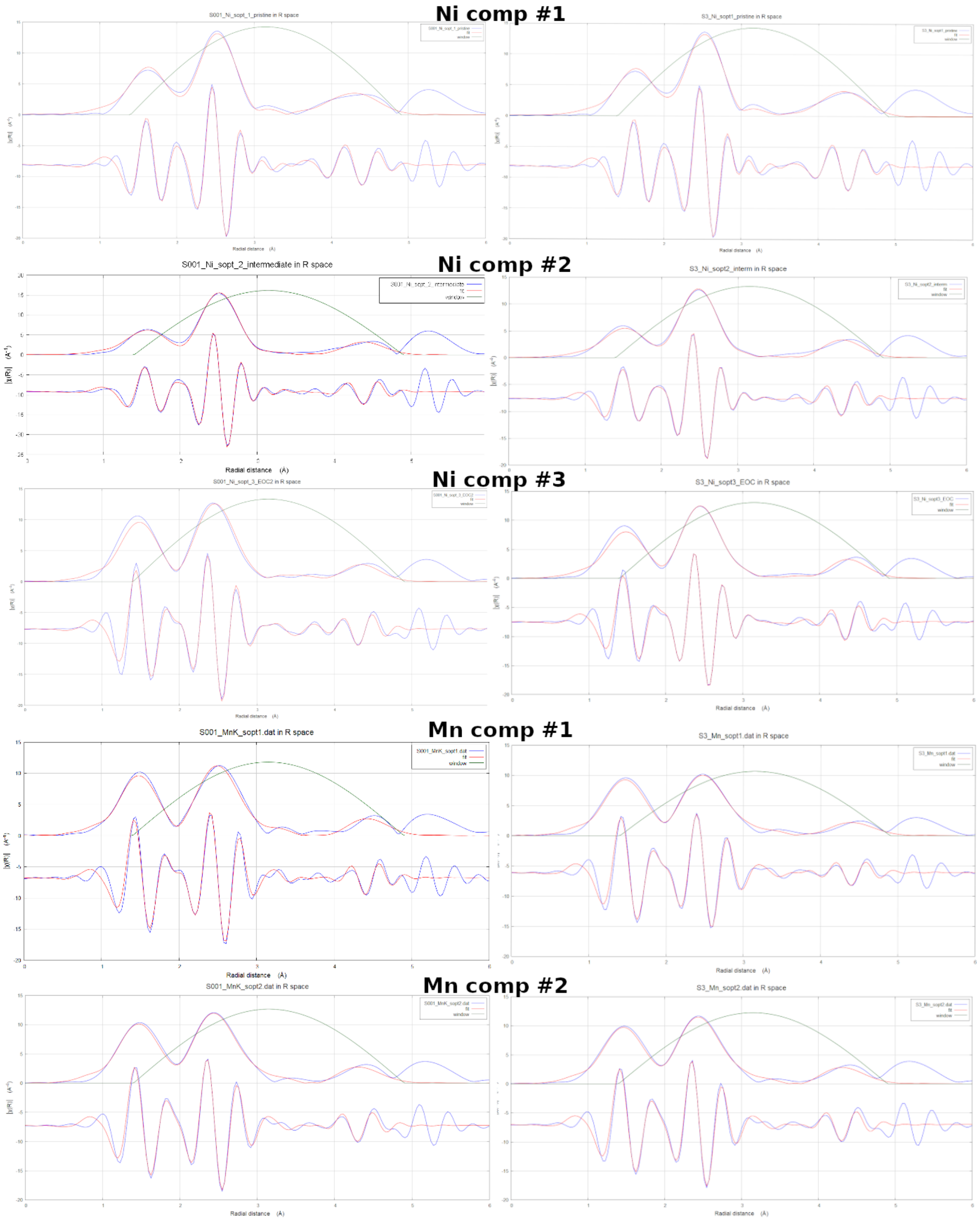

Figure S8: Fitted EXAFS spectra in R-space of pure MCR-ALS derived components for LNMO-D (left) and LNMO-O (right).

Table S3: TM K-edge EXAFS fitting parameters of MCR-ALS components for LNMO-D.

Component	Shell	N *	R_{fit} [Å]	σ^2 [Å]^2
MCR #1	Ni–O_1	6	2.051(7)	0.0064(9)
	Ni–TM	6	2.942(7)	0.0049(4)
	Ni–O_2	6	3.43(2)	0.004(3)
	Ni–O_3§	14	4.58(3)	0.008(2)
	Ni–O_4§	34	4.95(1)	
MCR #2	Ni–O_1	6	1.993(8)	0.008(1)
	Ni–TM	6	2.880(3)	0.0042(4)
	Ni–O_2	6	3.42(3)	0.008(4)
	Ni–O_3§	14	4.60(4)	0.011(7)
	Ni–O_4§	34	4.92(2)	
MCR #3	Ni–O_1	6	1.892(6)	0.0052(8)
	Ni–TM	6	2.818(4)	0.0060(5)
	Ni–O_2	6	3.79(5)	0.008(7)
	Ni–O_3§	16	4.58(3)	0.008(2)
	Ni–O_4§	32	4.84(2)	
MCR #1	Mn–O_1	6	1.901(6)	0.0053(8)
	Mn–TM	6	2.899(4)	0.0059(5)
	Mn–O_2	6	3.63(3)	0.008(4)
	Mn–O_3§	18	4.67(3)	0.007(2)
	Mn–O_4§	30	4.95(2)	
MCR #2	Mn–O_1	6	1.899(7)	0.004(1)
	Mn–TM	6	2.849(5)	0.0052(7)
	Mn–O_2	6	3.58(4)	0.008(6)
	Mn–O_3§	15	4.60(3)	0.008(3)
	Mn–O_4§	33	4.86(2)	

* Coordination numbers were evaluated and then kept fix for the presented fit.

+ Amplitude reduction factor was evaluated for pristine LNMO-D at the value of 0.95 and then kept fixed for all fits.

§ Only single scattering path contributions considered, coordination number should therefore be taken with care. Sum of coordination number constrained and mutual σ^2.

Table S4: TM K-edge EXAFS fitting parameters of MCR-ALS components for LNMO-O.

Component	Shell	N *	R_{fit} [Å]	σ^2 [Å]^2
	Ni–O_1	6	2.052(7)	0.0067(9)
	Ni–TM	6	2.906(4)	0.0051(4)
MCR #1	Ni–O_2	6	3.43(2)	0.003(2)
	Ni–O_3§	15	4.58(2)	0.007(1)
	Ni–O_4§	33	4.94(1)	
	Ni–O_1	6	1.954(7)	0.010(1)
	Ni–TM	6	2.859(3)	0.0058(4)
MCR #2	Ni–O_2	6	3.42(2)	0.010(4)
	Ni–O_3§	15	4.61(3)	0.007(2)
	Ni–O_4§	33	4.89(1)	
	Ni–O_1	6	1.903(6)	0.0069(9)
	Ni–TM	6	2.825(4)	0.0062(4)
MCR #3	Ni–O_2	6	3.45(4)	0.013(6)
	Ni–O_3§	17	4.60(2)	0.006(2)
	Ni–O_4§	31	3.86(1)	
	Mn–O_1	6	1.900(6)	0.0054(8)
	Mn–TM	6	2.879(4)	0.0064(6)
MCR #1	Mn–O_2	6	3.57(3)	0.009(5)
	Mn–O_3§	18	4.37(3)	0.008(3)
	Mn–O_4§	30	4.95(2)	
	Mn–O_1	6	1.899(8)	0.005(1)
	Mn–TM	6	2.852(5)	0.0057(7)
MCR #2	Mn–O_2	6	3.58(4)	0.009(6)
	Mn–O_3§	15	4.59(7)	0.008(3)
	Mn–O_4§	33	4.86(2)	

* Coordination numbers were evaluated and then kept fix for the presented fit.

+ Amplitude reduction factor was evaluated for pristine LNMO-O at 0.98, and then kept fixed for all fits.

§ Only single scattering path contributions considered, coordination number should therefore be taken with care. Sum of coordination number constrained and mutual σ^2.

References

(S1) Cabana, J.; Casas-Cabanas, M.; Omenya, F. O.; Chernova, N. A.; Zeng, D.; Whittingham, M. S.; Grey, C. P. Composition-Structure Relationships in the Li-Ion Battery Electrode Material $LiNi_{0.5}Mn_{1.5}O_4$. *Chem. Mater.* **2012**, *24*, 2952–2964.

(S2) Sushko, P. V.; Rosso, K. M.; Zhang, J.-G.; Liu, J.; Sushko, M. L. Oxygen Vacancies and Ordering of d-levels Control Voltage Suppression in Oxide Cathodes: the Case of Spinel LiNi0.5Mn1.5O4-δ. *Advanced Functional Materials* **2013**, *23*, 5530–5535.

(S3) Casas-Cabanas, M.; Kim, C.; Rodríguez-Carvajal, J.; Cabana, J. Atomic defects during ordering transitions in $LiNi_{0.5}Mn_{1.5}O_4$ and their relationship with electrochemical properties. *J. Mater. Chem. A* **2016**, *4*, 8255–8262.
